# Where Can a Threatened Pheasant Persist? Identifying Climate Change Refugia and Mapping Protection Gaps in China

**DOI:** 10.3390/ani16162475

**Published:** 2026-08-09

**Authors:** Min Li, Zhengliang Fan, Lianxiao Wang, Yudong Wang, Rong Dong, Zhengwang Zhang, Xiaoping Yu

**Affiliations:** 1 Shanxi Key Laboratory of Coordinated Management and Control for Environmental Quality, School of Environment and Resources, Taiyuan University of Science and Technology, Taiyuan 030024, China; limin19910828@163.com (M.L.); fanfanzl1006@163.com (Z.F.); 18835246982@163.com (L.W.); 2 Shanxi Provincial State-Owned Forest Farm and Seedling Work Station, Shanxi Forestry and Grassland Bureau, Taiyuan 030012, China; shanxitianbao2020@163.com; 3 Research Center for Qinling Giant Panda, Shaanxi Academy of Forestry, Xi’an 710400, China; donglittle@sohu.com; 4 Key Laboratory for Biodiversity Science and Ecological Engineering, College of Life Sciences, Beijing Normal University, Beijing 100875, China; 5 College of Life Sciences, Shaanxi Normal University, Xi’an 710119, China

**Keywords:** brown eared pheasant, climate change, GAP analysis, Marxan model, species distribution models

## Abstract

Climate change and human activities are widely recognized as major drivers of global biodiversity loss. The Brown Eared Pheasant (*Crossoptilon mantchuricum*), a ground-dwelling avian species unique to China, is confined to a highly fragmented range and faces persistent long-term threats. To devise robust and site-specific preservation strategies, it is imperative to elucidate the species’ sensitivity to climate change and pinpoint high-priority protection areas. In this study, we integrated field surveys, ensemble species distribution modelling, Marxan systematic conservation planning, and GAP analysis to assess habitat suitability changes under future climate scenarios and identify conservation gaps for the Brown Eared Pheasant. Our results demonstrate that the species’ distribution is primarily driven by annual temperature range, precipitation in the coldest and wettest quarters, and elevation, underscoring its highly restricted climatic niche. Based on current climatic conditions, highly suitable habitats are primarily distributed across specific mountainous regions: Hebei Province (Xiaowutai Mountains), Shanxi Province (the Taihang, Lvliang, and Taiyue mountain systems), Shaanxi Province (Huanglong Mountains), and northwestern Beijing (the Donglingshan area). Simulations conducted across prospective climatic scenarios suggest a continued decline in suitable habitat, which becomes increasingly restricted to higher latitudes and mountainous regions. According to our GAP analysis, current protected area networks fail to encompass 72.27% of the priority conservation zones identified, with significant gaps in the southern Lvliang, Taiyue, and Huanglong Mountains. The outcomes of this research establish a robust empirical foundation with which to inform prospective preservation strategies and habitat stewardship for the Brown Eared Pheasant.

## Simple Summary

Climate change and habitat loss threaten many wildlife species, particularly those with small ranges and specific habitat requirements. The Brown Eared Pheasant (*Crossoptilon mantchuricum*) is a unique bird species in China that survives in fragmented mountain forests, and it is sensitive to environmental changes. In this study, we aimed to determine how future climate change may influence its habitat and identify areas that require greater protection. By combining field surveys and spatial analyses, we assessed changes in suitable habitats and examined whether current protected areas can provide enough coverage. We found that temperature changes, rainfall patterns, and elevation strongly influence where suitable habitats occur. Future climate change is expected to reduce suitable habitats and increase the separation of remaining populations, especially in some current core areas. Many important habitats identified in this study are not currently protected. These findings provide useful information for improving habitat protection, maintaining connections between populations, and developing effective conservation strategies for the long-term survival of the Brown Eared Pheasant.

## 1. Introduction

Habitat loss and degradation, driven primarily by climate change, are projected to be major drivers of biodiversity decline in the coming decades [1,2]. According to the IPCC’s Sixth Assessment Report (AR6), between 2011 and 2020, global mean surface temperatures rose by 1.1 °C compared to late 19th-century pre-industrial levels [3]. Alterations in thermopluviometric dynamics concurrently dictate the structural reconfiguration of natural habitats and exert profound impacts on ethology, physiological mechanisms, and population demographics across a wide array of animal lineages [4,5]. With the intensification of global warming, some species are shifting their ranges toward higher latitudes or elevations, while others are undergoing physiological adaptations to survive in situ [6,7]. Particularly for vulnerable taxa with limited distributions, forecasting how suitable habitats will shift across various climate projections is crucial for formulating evidence-based and actionable management plans [8,9].

Quantifying where species can persist under environmental change has become a cornerstone of modern conservation planning. Species Distribution Models (SDMs)—frequently termed habitat suitability models—provide a quantitative framework for linking species occurrence data with environmental factors [10]. Over the past two decades, SDMs have been widely used across disciplines such as biogeography, conservation biology, and climate change impact assessment. More than a dozen modeling algorithms are routinely employed, though their predictive performance varies considerably [11,12]. However, studies have shown that the performance of individual models can be highly sensitive to variations in input data, resulting in relatively low stability and reproducibility [13,14]. To overcome these methodological constraints, the BioMod2 framework was established—an R environment package tailored specifically for generating ensemble species distribution models [15]. Subsequent comparative studies have shown that ensemble approaches implemented through BioMod2 consistently achieve higher discrimination and calibration accuracy than single-model strategies [16].

Systematic conservation planning (SCP) is a structured approach to ecologically based zoning, enabling the design of conservation networks that harmonize biological priorities with human development demands [17,18]. Among the most widely used tools in SCP is Marxan, which employs a simulated-annealing algorithm to identify cost-efficient portfolios of planning units while incorporating socioeconomic constraints, boundary length minimization, and biodiversity representation targets [19]. Complementing this approach, GAP analysis utilizes GIS frameworks to map the distribution of key ecological elements, such as distinct vegetation communities and endangered fauna, revealing critical deficiencies in the representation of these features within established conservation estates [20]. The combination of GAP analysis with Marxan forms a robust decision-support framework that enhances conservation planning across landscape and regional scales [21].

**Figure 1:**
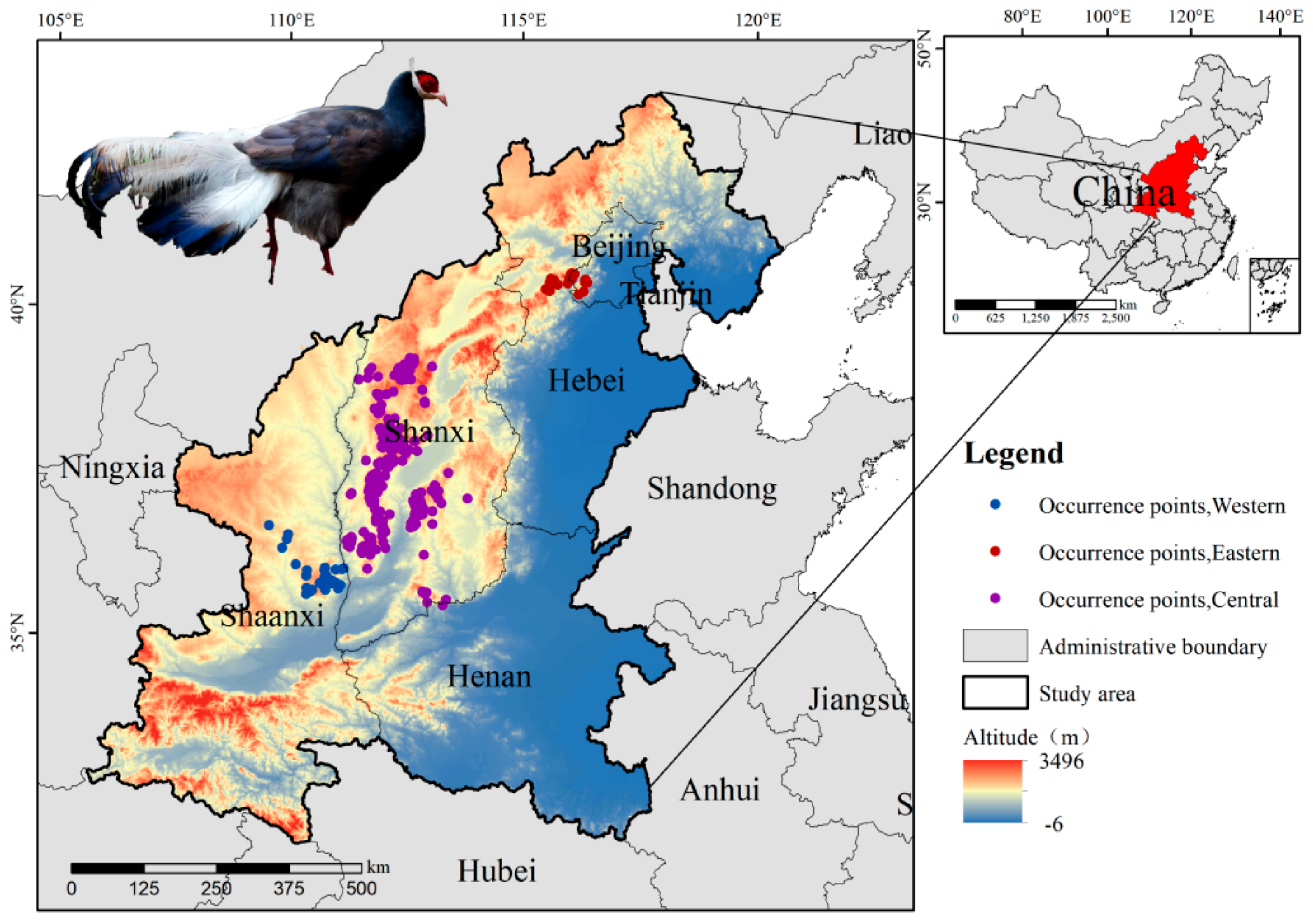
Study area and the occurrence points of the Brown Eared Pheasant.

The Brown Eared Pheasant (*Crossoptilon mantchuricum*), an endemic galliform species in China with significant ecological, cultural, and economic value, persists in three geographically isolated populations confined to fragmented montane habitats across Northern China. The easternmost population occupies the Xiaowutai and Donglingshan massifs across Hebei and northwestern Beijing; the central population is primarily found within Shanxi’s Lvliang, Taiyue, and Zhongtiao ranges, though recent evidence suggests an outward dispersal into the southern Taihang Mountains of Henan [22]; and the western population is confined to the Huanglong mountainous region of Shaanxi (Figure 1) [23,24]. Intensive logging, agricultural expansion, and hunting through the ages have led to a decline in the total population, now estimated at only 5000–15,000 individuals [25]. Although the species was reclassified as being of Least Concern in the 2025 IUCN assessment, its narrow ecological niche, limited flying ability, and dependence on mid-elevation forest understory make it particularly vulnerable to climate change [26,27,28]. Despite numerous studies on its habitat suitability and population distribution, a comprehensive analysis of climate-driven habitat shifts and corresponding systematic conservation planning has not been carried out [22,23,24,25].

In this study, we utilized an ensemble SDM framework to project the current and future climatic niches for the Brown Eared Pheasant across various climate scenarios. To identify priority conservation areas, we generated planning units and conservation feature layers for use as inputs in the Marxan model. Furthermore, a GAP analysis was conducted to quantify representation gaps within the existing protected area network. The primary objectives of this research are threefold: (i) determine the critical environmental drivers shaping the geographic range of the Brown Eared Pheasant; (ii) predict potential suitable habitats under contemporary and prospective climatic scenarios and assess associated spatiotemporal changes; and (iii) delineate spatial conservation priorities and evaluate gaps within the current protected area network.

## 2. Methods

### 2.1. Study Area

The geographical focus of this research encompasses the central portion of North China (31°21′~42°58′ N, 105°56′~119°20′ E) (Figure 1). Administratively, the study area covers Beijing as well as the provinces of Hebei, Shanxi, Shaanxi, and Henan. Encompassing approximately 732,000 km^2^, the region features diverse topographical conditions, including mountain ranges, rivers, and valleys, which may restrict the dispersal of the Brown Eared Pheasant. The climate is warm-temperate and semi-humid continental, featuring hot, humid summers alongside cold, arid winters. The mean annual temperature ranges from 8 to 13, with yearly precipitation ranging from 300 to 1000 mm. Topographically, the landscape transitions from expansive eastern lowlands to rugged mountainous terrain across the northern and western frontiers, with elevations ranging from 0 to 3643 m. The natural vegetation predominantly consists of warm-temperate deciduous broadleaf forests [29]. Within this region, the Brown Eared Pheasant is considered a sensitive indicator of environmental change. It fulfills key ecological roles and has been recognized as both a flagship and umbrella species [24,30].

### 2.2. Species Occurrence Data

Occurrence records for the Brown Eared Pheasant were gathered from three primary sources: (i) field and camera-trapping surveys conducted by the Shanxi Provincial Forestry and Grassland Administration and Hebei Xiaowutaishan National Nature Reserve from 2022 to 2024; (ii) the Global Biodiversity Information Facility occurrence records from the past five years (GBIF database, https://www.gbif.org/, accessed 30 December 2024); and (iii) peer-reviewed publications. All occurrence points were georeferenced using locality descriptions and detailed satellite imagery from Google Earth. To mitigate the risk of model overfitting caused by spatial autocorrelation [31,32], we applied spatial thinning to all the occurrence records prior to modeling using the “spThin” package version 0.2.0 in R version 4.4.1 [33,34]. Specifically, a single occurrence point was randomly kept within each 1 km × 1 km grid cell, yielding a final dataset consisting of 265 spatially independent records for subsequent habitat modelling.

### 2.3. Environmental Variables

We initially considered 19 climatic variables (Bio1–Bio19), elevation (DEM), the Terrain Ruggedness Index (TRI), and road density (RD) as potential predictors. All data sources used in this study are detailed in Table 1. Historical climate variables representing the baseline climatic conditions (1970–2000) were obtained from the WorldClim v2.1 bioclimatic dataset. Future climatic variables for the mid-century (2041–2060) and end-of-century (2081–2100) periods were acquired from the BCC-CSM2-MR model, operating within the Coupled Model Intercomparison Project Phase 6 (CMIP6) framework. This model has been widely applied in ecological niche modeling studies and demonstrated good performance in regional climate simulations and climate change studies in China. These forecasts incorporate three distinct Shared Socioeconomic Pathway scenarios: SSP126, SSP245, and SSP585 [35,36]. All environmental layers from different sources were resampled to a common spatial resolution of 1 km before modeling was conducted. To preclude algorithmic overfitting induced by predictor multicollinearity, a Pearson correlation matrix was constructed for all environmental variables (Figure S1); only factors exhibiting a correlation coefficient below 0.8 were retained for the final modeling phase [37]. The final set of predictors selected for subsequent modeling comprised annual temperature range (Bio7), precipitation in the wettest month (Bio13), isothermality (Bio3), precipitation in the coldest quarter (Bio19), maximum temperature in the warmest month (Bio5), DEM, TRI, and RD.

### 2.4. Construction and Evaluation of SDMs

Distribution modeling for the Brown Eared Pheasant was executed via the “Biomod2” package version 4.2-6-2 in R version 4.4.1, incorporating ten distinct algorithms: Random Forest (RF), Maximum Entropy (MaxEnt), Artificial Neural Networks (ANNs), Generalized Boosted Models (GBMs), Classification Tree Analysis (CTA), Generalized Linear Models (GLMs), Generalized Additive Models (GAMs), Multivariate Adaptive Regression Splines (MARS), Flexible Discriminant Analysis (FDA), and Surface Range Envelope (SRE). For the MaxEnt model, we conducted hyperparameter tuning via the “ENMeval” package, setting the regularization multiplier (RM) to 0.5 alongside a linear and quadratic (LQ) feature class configuration. Default parameter settings were adopted for all other algorithms [38]. To ensure predictive reliability, the occurrence data were randomly split: 25% were withheld for independent validation, while the remaining 75% were allocated for model calibration. To improve model robustness, 2000 random pseudo-absence points were created, and the complete modeling process was iterated 5 times [15].

Higher AUC and TSS values indicate better predictive accuracy of a model. The final ensemble model was built on the basis of the highest-performing algorithms (AUC ≥ 0.80) selected from the six candidate models. This ensemble model was then projected across the study area to produce a continuous habitat suitability surface, with values scaled from 0 (unsuitable) to 1000 (highly suitable). The output suitability layers were loaded into ArcGIS 10.2 for visualization and subsequent spatial analysis. Using the Natural Breaks (Jenks) classification method, we categorized habitat-suitability projections into four classes: unsuitable (class 1), low suitability (class 2), moderate suitability (class 3), and high suitability (class 4) [39]. Additionally, response curves derived from the ensemble model were analyzed to investigate the relationships between key environmental variables and habitat suitability for the Brown Eared Pheasant.

### 2.5. Changes in the Spatial Patterns of Suitable Habitats for This Species

Under climate change, the spatiotemporal dynamics of viable habitats can be categorized into three distinct trajectories: stable, contracting, and expanding areas [40,41]. To assess habitat changes for the Brown Eared Pheasant, unsuitable areas (class 1) were coded as 0, while low-, moderate- and high-suitability areas (classes 2–4) were coded as 1. The resulting binary maps were compared between the current period and the future climate scenario. In this framework, a transition from 0 to 1 indicates range expansion, a shift from 1 to 0 represents habitat shrinkage, and 1 to 1 signifies stable suitability. The habitat expansion and contraction were calculated relative to the total suitable habitat area under each corresponding future climate scenario. Finally, the resultant matrix data were converted into spatial attributes and handled using ArcGIS 10.2 in order to map the spatio-temporal dynamics of suitable habitat distributions.

### 2.6. Marxan Model Construction

Marxan version 2.43 is a frequently applied spatial prioritization software that utilizes the simulated annealing algorithm to identify cost-effective conservation solutions under predefined constraints [42,43]. In this study, we applied Marxan to designate critical protection zones for the Brown Eared Pheasant, using a 10 km ×10 km grid as the spatial planning unit to cover the study area. Four key environmental and anthropogenic variables were incorporated as protection costs within each planning unit: Human Activity Intensity (HAI), the Normalized Difference Vegetation Index (NDVI), the Terrain Ruggedness Index (TRI), and Elevation (DEM) [44]. The HAI is a comprehensive indicator reflecting the extent of human impact on terrestrial surfaces, integrating metrics such as road density, population density, global impervious surface area proportion, and the Landscape Development Intensity Index (LDI) [45,46]. The computational formula is presented below:

$$HAI=\sum_{i=1}^{n}X_{i}\times W_{i}$$

In this formula, *X_i_* denotes the normalized value for the *i*-th indicator, and *W_i_* represents its allocated weight. The weights were computed via the entropy-weighting technique [47]. The computational procedure involved the following steps: (i) standardizing each indicator using min–max normalization across all planning units; (ii) calculating the entropy value for each index; and (iii) deriving the entropy weight. Since higher values indicate greater human disturbance, all indicators were treated as positive indicators. The resulting calculated weights for these four variables are as follows: population density, 0.3494; global impervious surface area percentage, 0.1263; road density, 0.2653; and Landscape Development Intensity Index (LDI), 0.2590.

The LDI metric serves as a proxy for potential anthropogenic disturbance calculated using land-cover data. It was computed in accordance with the methodology developed by Brown and Vivas [48], utilizing the LDI coefficients listed in Table S1. The LDI value for a given spatial unit is calculated as follows:

$$LDI_{total}=\sum \%{LU}_{i}\times {LDI}_{i}$$

where *LDI_total_* represents the total Landscape Development Intensity score of the spatial unit; %*LU_i_* denotes the percentage of the region covered by the *i*-th land cover class; and *LDI_i_* refers to the corresponding LDI coefficient allocated to the *i*-th land cover category.

To guide the spatial prioritization of key habitats, we established conservation targets specifying the proportion of each habitat suitability class to be included in Marxan’s final solution. This approach allowed us to evaluate the optimal conservation outcomes’ sensitivity to target definition. In alignment with Target 3 of the Kunming–Montreal Global Biodiversity Framework [49], we set a 30% conservation target for high-suitability habitats. For moderate-suitability habitats, a lower target, 15%, was used to retain representative complementary habitats. The Species Penalty Factor (SPF) and Boundary Length Modifier (BLM) helped optimize the spatial design, ensuring the target species were protected within well-connected and compact boundaries. Sensitivity testing was performed via the generation of sensitivity curves to identify an optimal combination of SPF and BLM values, resulting in the selection of a BLM parameter of 0.01 alongside an SPF parameter of 1.1. The model was run 100 times, with each run consisting of 1,000,000 simulated annealing iterations, in order to identify the best configuration of the planning units [17,50]. Finally, we used ArcGIS to visualize the outputs and design a spatial conservation layout for the Brown Eared Pheasant.

### 2.7. GAP Analysis of Protected Areas for the Brown Eared Pheasant

**Figure 2:**
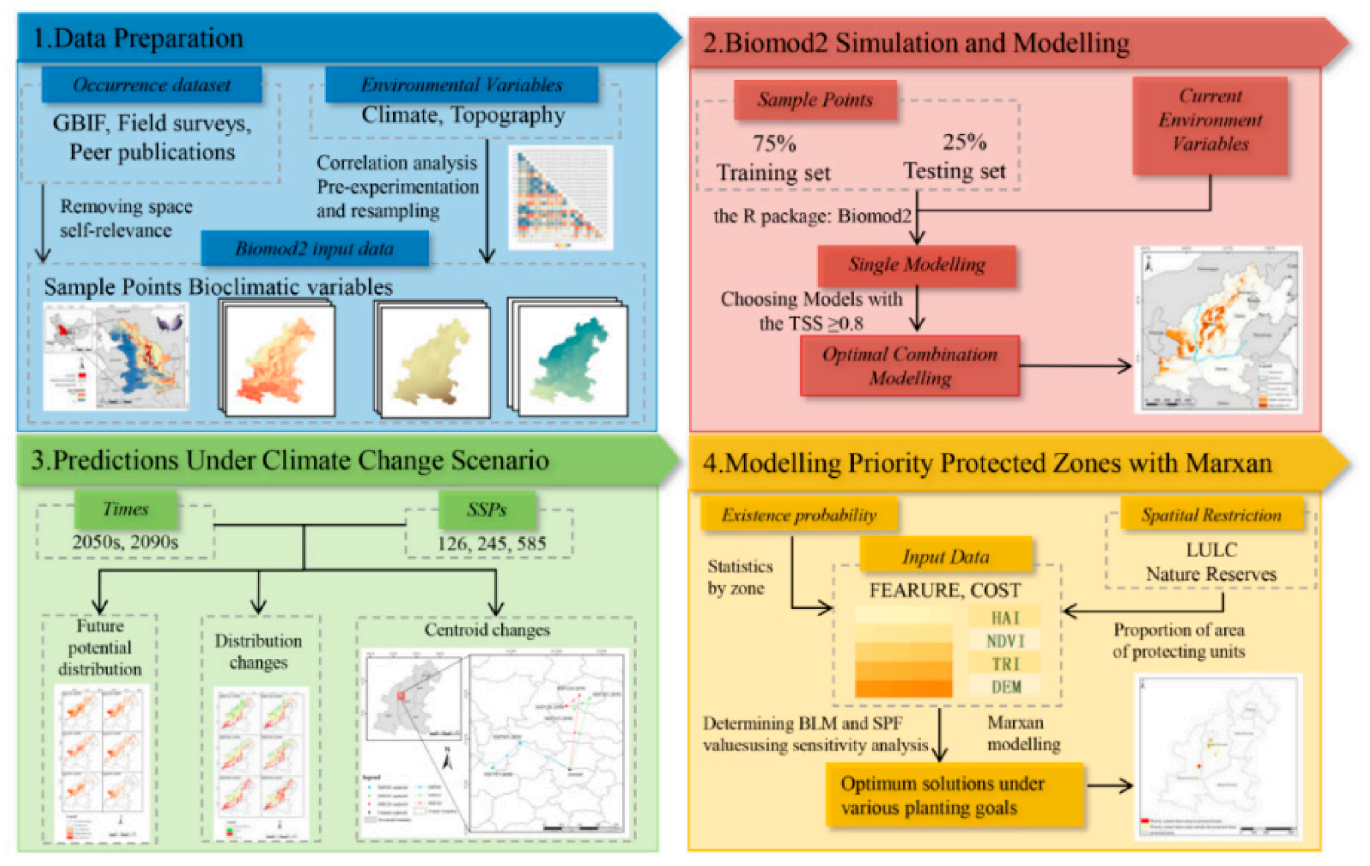
Conceptual and methodological flowchart of this study.

To measure conservation effectiveness, a GAP analysis was applied to highlight the shortfall between established targets and the actual coverage of current nature reserves. Areas of high conservation priority that fall outside established reserves are considered conservation gaps [51]. In this study, we incorporated 21 nature reserves located within the study area (Table S2). To assess the current conservation state of the Brown Eared Pheasant, we overlaid the final Marxan solution layer with the spatial perimeters of established protected zones in ArcGIS. The resulting map of the conservation gap highlights unprotected optimal environments supporting the Brown Eared Pheasant, providing a scientific basis for developing targeted regional conservation strategies. The analytical workflow is summarized in Figure 2.

## 3. Results

### 3.1. Model Accuracy Evaluation

All ten individual models were successfully developed, and their accuracy metrics are shown in Table 2. GBM yielded the highest evaluation scores (AUC = 0.8840; TSS = 0.8004) and remained highly stable across runs. Conversely, the FDA approach produced the poorest predictions, with an AUC of 0.7582 and a TSS of 0.7042. The ensemble model, which integrated the six best-performing individual models (GAM, GBM, GLM, MaxEnt, RF, and SRE), outperformed all individual models, attaining an AUC of 0.9520 and a TSS of 0.8328. The results suggest it has high predictive accuracy in identifying suitable habitat for the Brown Eared Pheasant. Consequently, all subsequent analyses in this study were conducted using the output from the ensemble model.

### 3.2. Importance of Environmental Variables

**Figure 3:**
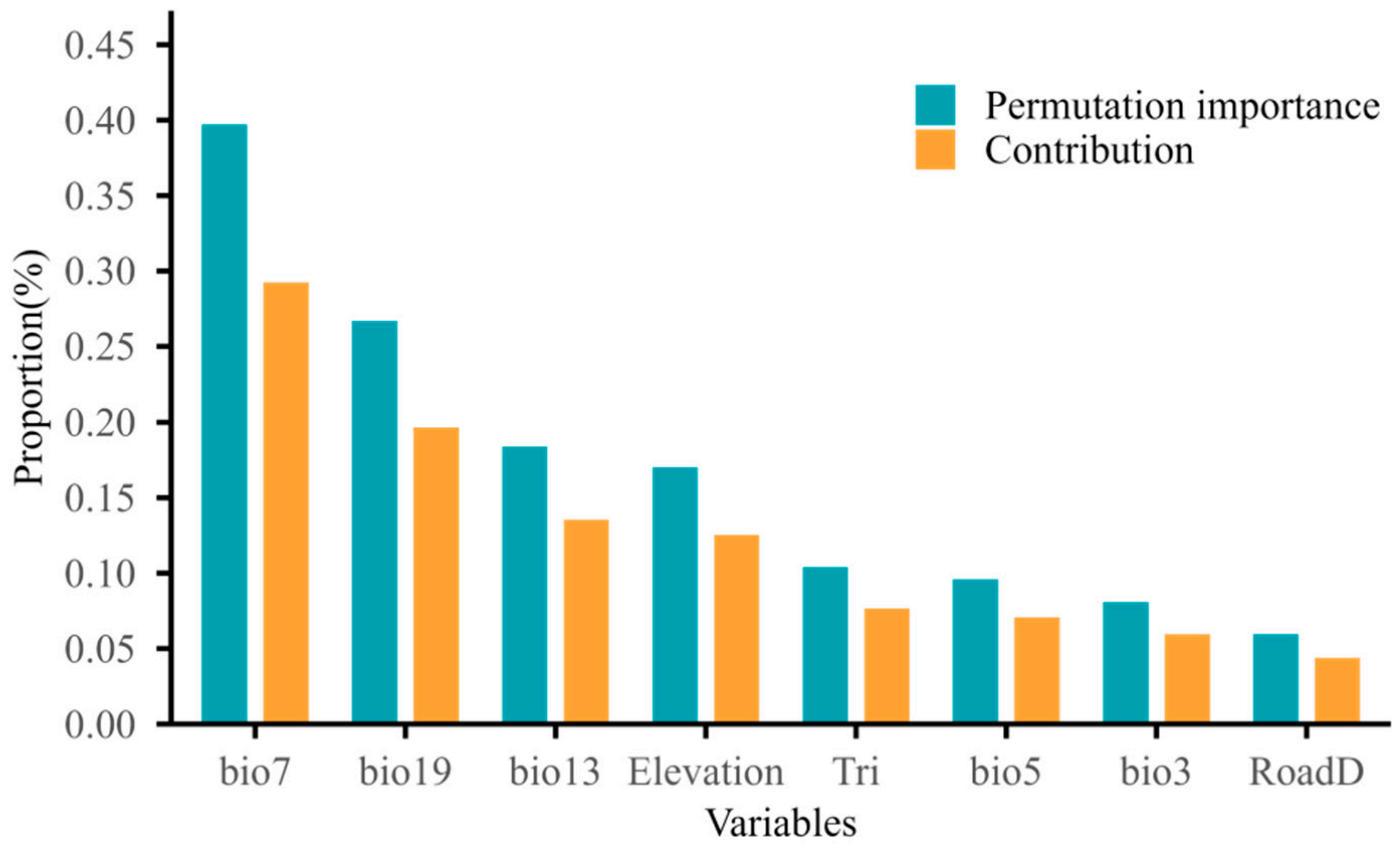
Contribution rates and permutational importance of the environmental variables.

Whereas a variable’s contribution denotes its broad impact on modeled habitat suitability, permutation importance quantifies the reduction in predictive power that occurs when the variable’s values are randomly permuted. Comparative analysis revealed that the annual temperature range (Bio7) accounted for the highest contribution (29.23%), underscoring its critical role in shaping the distribution of the Brown Eared Pheasant. This was followed by precipitation in the coldest quarter (Bio19), at 19.66%; precipitation in the wettest month (Bio13), at 13.55%; and elevation (DEM), at 12.52% (Figure 3). Together, these four variables—each exceeding 10% in terms of contribution—accounted for 74.96% of the total influence, indicating they are the primary environmental determinants of habitat suitability for the Brown Eared Pheasant.

**Figure 4:**
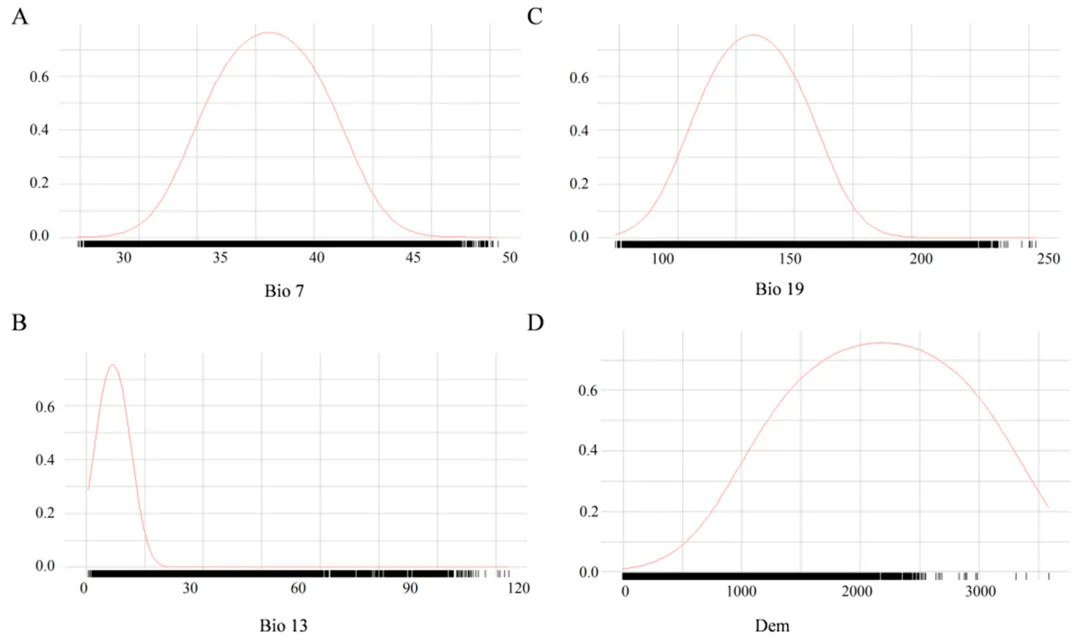
Response curves for the dominant environmental variables: (**A**) temperature annual range (Bio7); (**B**) precipitation of coldest quarter (Bio19); (**C**) precipitation of wettest month (Bio13); (**D**) elevation (DEM).

The marginal response curves, which demonstrate how the top four environmental predictors govern the occurrence probability of the Brown Eared Pheasant, are presented in Figure 4. Areas with environmental conditions with predicted occurrence probabilities exceeding 0.6 are considered highly suitable habitats for the Brown Eared Pheasant. Within the displayed intervals, the optimal ranges of the key environmental factors are as follows: an annual temperature range of 35–40 °C, a precipitation range of 8–15 mm in the coldest quarter, a precipitation range of 110–160 mm in the wettest month, and an elevation range of 950–2600 m.

### 3.3. Geographical Distribution of the Brown Eared Pheasant Under Current and Future Climate Scenarios

**Figure 5:**
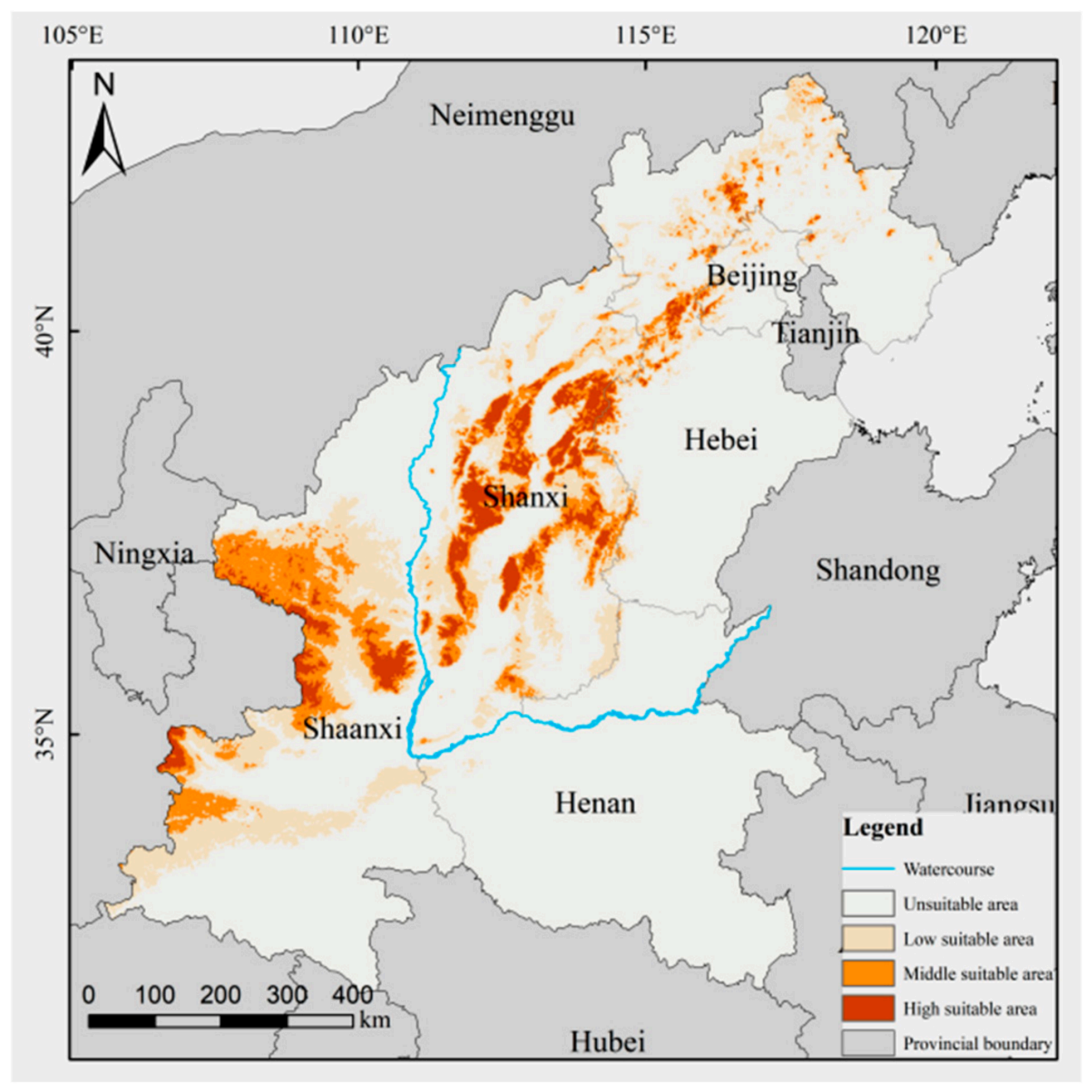
Potential distribution of the Brown Eared Pheasant in the study area under the baseline climate situation.

Under current climatic conditions, the area of the total favorable environment supporting the Brown Eared Pheasant was calculated to be 18.70 × 10^4^ km^2^ (Figure 5). The corresponding areas are primarily located across the Xiaowutai ranges of Hebei; the Taihang, Lvliang, and Taiyue mountain chains of Shanxi; the Huanglong regions of Shaanxi; and the northwestern mountainous areas in Beijing. In these regions, the high-suitability areas cover approximately 2.70 × 10^4^ km^2^, representing 3.69% of the entire study region. Zones of medium suitability, which are spatially contiguous with the high-suitability core zones, occupy 5.40 × 10^4^ km^2^, constituting 7.37% of the overall research landscape. Conversely, low-suitability regions exhibit the most extensive distribution, covering 10.59 × 10^4^ km^2^, or 14.34% of the total study area.

**Figure 6:**
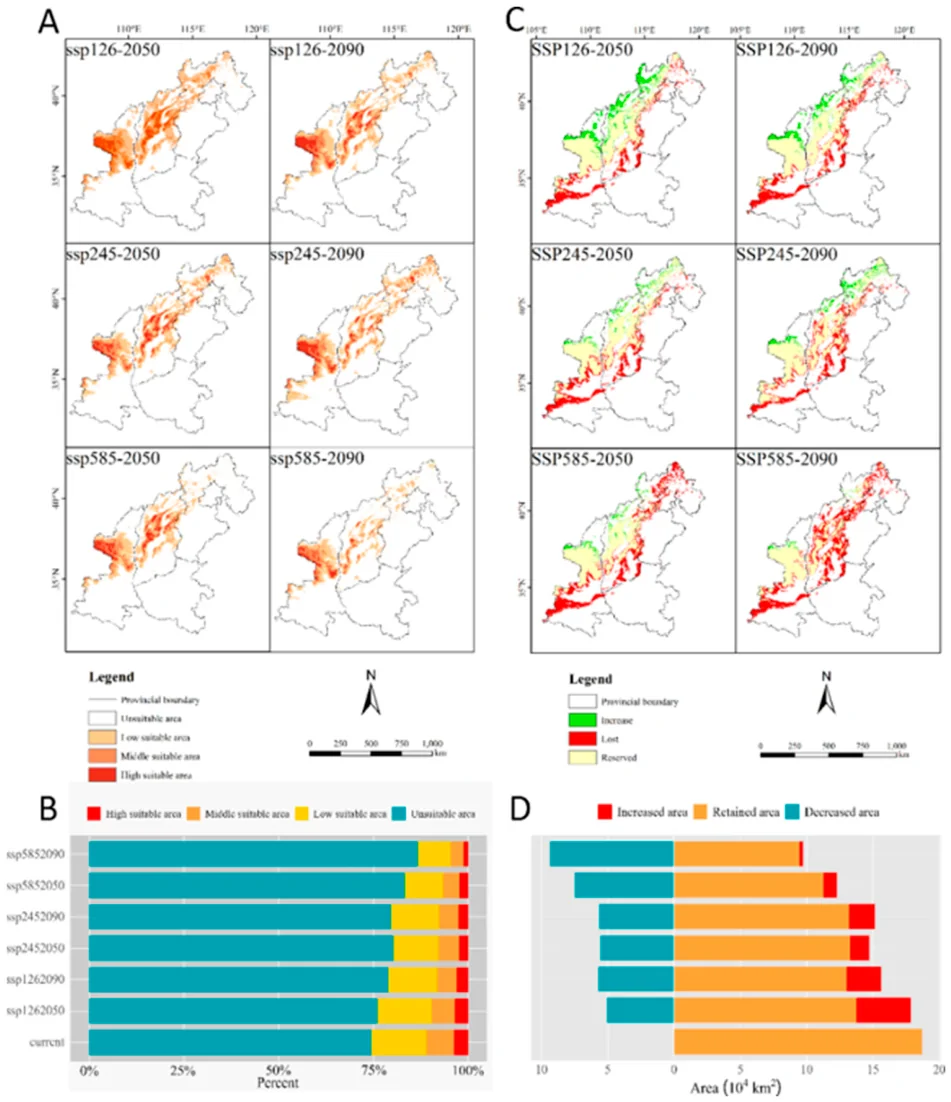
Distribution of and changes in potential suitable habitat for Brown Eared Pheasant under future climate scenarios: (**A**) predicted spatial distribution under future climate scenarios; (**B**) area changes in low-/middle-/high-suitable habitats under future climate scenarios; (**C**) spatial shifts in potential habitat from current to future conditions; and (**D**) total area changes in suitable habitat over time.

We mapped the future suitable habitats for the Brown Eared Pheasant by applying projected shifts in bioclimatic variables, as depicted in Figure 6. Compared to the current baseline, our projections indicate that the overall habitable space will shrink across the majority of future climate scenarios, though a degree of resilience is evident under the SSP245 scenario. Specifically, under SSP245, the area of high-suitability habitat declines from 2.70  × 10^4^ km^2^ (current) to 1.76  × 10^4^ km^2^ (2050s), followed by a slight recovery to 1.84 ×10^4^ km^2^ by the 2090s. In contrast, the high-emission SSP585 scenario leads to a pronounced contraction in high-suitability areas, shrinking from 1.71 × 10^4^ km^2^ in the 2050s to only 0.90 ×10^4^ km^2^ by the 2090s.

Integrating current distributional data with modeled projections under various climatic scenarios, we quantified subsequent spatial changes in potential favorable zones for this target species (Figure 6C). Relative to the current baseline, the species is projected to retain the largest proportion of its habitat unchanged across all scenarios. However, the percentage of habitat loss consistently exceeds that of expansion, though the magnitude of change varies among climate scenarios. Suitable habitat expansion occurs primarily across the northwestern sector of the research zone, including the northern foothills of both the Taihang and Lvliang mountains, the Xiaowutai mountains, and parts of northwestern Beijing. Expansion rates range from 2.39% to 21.92%, where minimum values were recorded during the SSP126-2050s projection, peaking within SSP585-2090s, indicating a general northward redistribution of habitat suitability. In contrast, the most substantial habitat loss is projected to be in the central and southern mountainous regions of Shanxi Province, particularly within the mid-elevation zones of the Lvliang Mountains and the southern Taiyue Mountains. These areas, which are currently the core habitat for the Brown Eared Pheasant because of their lower latitudes and elevation, are projected to experience extensive degradation, reaching a 75.5% reduction under the SSP585-2090s scenario.

### 3.4. Shifts in the Centroid of Suitable Habitat Under Climate Change Scenarios

**Figure 7:**
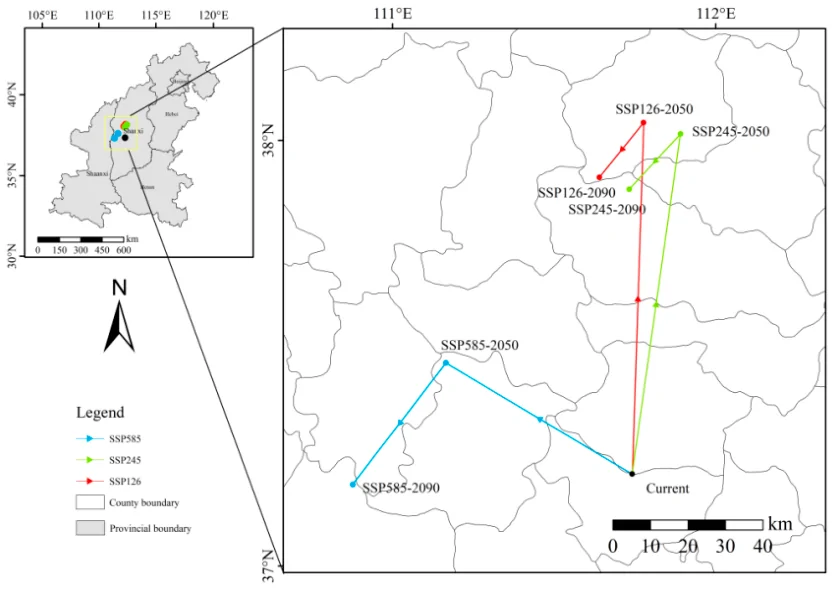
Geographic shifts in the centroid of the habitats predicted to be potentially suitable for the Brown Eared Pheasant under different climate scenarios.

The spatial displacement of the habitat’s geographic core (centroid) under various projected climate conditions was quantified, the results of which are depicted in Figure 7. Across all scenarios, the geographic centroid of suitable areas remains within Shanxi Province, whereas both the direction and distance of centroidal shifts vary with emission intensity. Under the SSP126 and SSP245 scenarios, the centroid shifts consistently northeastward by the 2050s, moving 91.84 km and 89.86 km, respectively, toward higher latitudes. By the 2090s, however, it experiences a slight southwestward retreat amounting to 18.70 km and 20.15 km under the same scenarios. In contrast, under the high-emission SSP585 scenario, the centroid initially moves 58.17 km northwest—from 111.64° E/37.16° N to 111.10° E/37.45° N—by the 2050s. Subsequently, it shifts an additional 40.72 km southwest, reaching 95.08° E/31.31° N by the 2090s.

### 3.5. GAP Analysis

**Figure 8:**
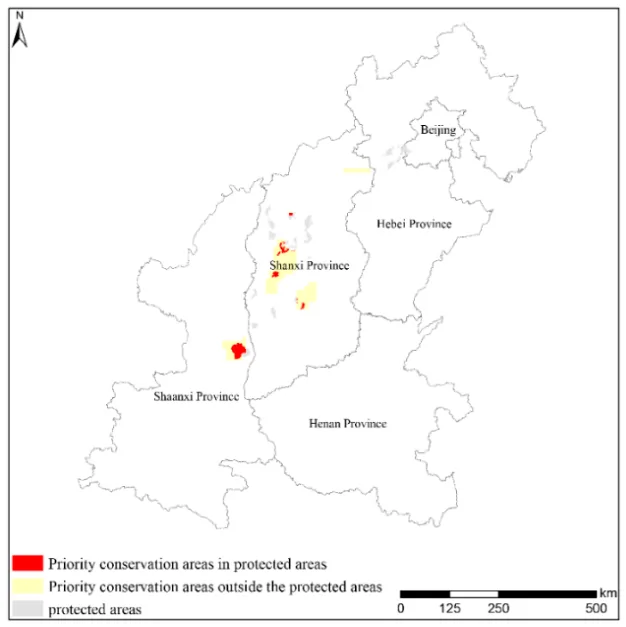
Conservation gaps for the Brown Eared Pheasant between identified priority areas and existing nature reserves.

High-priority conservation zones for the Brown Eared Pheasant were delineated utilizing Marxan software and subsequently intersected with current protected area networks to visually assess spatial conservation deficits (Figure 8). The results reveal that only 27.73% of these priority areas fall within current nature reserves, while the remaining 72.27% lack formal protection, highlighting significant gaps in the current conservation network. Furthermore, the strong spatial agreement between the Marxan-derived priority zones and the habitats predicted to be suitable by the ensemble model indicates that the identified conservation priorities were mainly concentrated within areas of high habitat suitability.

## 4. Discussion

### 4.1. Dominant Environmental Drivers Shaping the Potential Distribution of the Brown Eared Pheasant

Identifying the environmental factors that shape a species’ ecological niche is fundamental to effective conservation [52]. Studies have investigated the environmental preferences and habitat quality for the Brown Eared Pheasant at localized [30,53] or partial landscape scales [25,27,54], yet none have integrated field data encompassing all known populations. In this work, we applied an ensemble modeling framework to evaluate how climate, topography, and human activities drive current habitat suitability for the Brown Eared Pheasant. Comparable evidence from the Qinghai–Tibet Plateau indicates that climatic factors, particularly precipitation, interact with human disturbance to shape mammal distributions and the spatial configuration of priority conservation areas [55].

These results reveal how annual temperature range (Bio7) and two precipitation-related factors—precipitation in the wettest month (Bio13) and precipitation in the coldest quarter (Bio19)—are essentially the dominant environmental drivers shaping the species’ distribution. This finding underscores the Brown Eared Pheasant’s sensitivity to both thermal stability and moisture availability. Extreme annual thermal oscillations are likely to increase energy expenditure for thermoregulation during seasonal extremes, while inadequate cold-season precipitation may limit vegetation growth and reduce the availability of seeds and fruits in late winter—a critical period for galliform foraging. Conversely, high summer rainfall can elevate the risk of disease and parasite proliferation and reduce nesting success in this ground-nesting bird. Elevation (950–2600 m) emerged as another key factor shaping habitat suitability, a finding consistent with earlier studies [25,27,54], further reflecting the species’ adaptation to mid-elevation montane environments. Similar patterns have also been reported in relation to other montane pheasants, including the Golden Pheasant (*Chrysolophus pictus*) and Blue Eared Pheasant (*Crossoptilon auritum*), for which climatic variables and elevation are important determinants of habitat suitability [56,57]. These consistencies suggest that the environmental requirements of the Brown Eared Pheasant are broadly consistent with those of other forest-dependent galliforms.

### 4.2. Effect of Climate Change on the Geographical Distribution of the Brown Eared Pheasant

Changes in species ranges are a common ecological consequence of climate change and largely driven by global warming [5]. Quantifying how climate change alters potential species distributions is essential for designing conservation strategies aimed at sustaining ecosystem equilibrium [58]. According to our combined models, the main challenge the Brown Eared Pheasant will have to respond to regarding future climate change is the shrinking of its habitable area.

Under the high-emission pathway (SSP585), the area of high-suitability habitat is projected to decline by 66.7%, shrinking to only 9000 km^2^ by the 2090s, while the total suitable area will decrease by more than 30% (Figure 6B,D). These reductions are linked to continued greenhouse gas accumulation, elevated mean temperatures, and a rising frequency of severe climatic phenomena, including droughts and heavy precipitation, all of which contribute to the degradation of mid-elevation forest ecosystems. In contrast, under the intermediate-emission scenario (SSP245), the high-suitability areas show a U-shaped trajectory, declining to a minimum of 17,600 km^2^ by the 2050s before partially recovering to 18,400 km^2^ by the 2090s, underscoring the conservation benefits of mid-century emission mitigation.

Future climate scenarios indicate not only a reduction in the total suitable area for the Brown Eared Pheasant but also significant changes in its spatial configuration. Habitat gains are largely concentrated in northern and mountainous regions, including the northern foothills of the Taihang and Lvliang Mountains, the Xiaowutai Mountains, and northwestern Beijing. In contrast, reductions are concentrated in current core habitats in central and southern Shanxi. Moreover, the rate of habitat loss substantially exceeds the rate of expansion: gains range from 2.4% (SSP126-2050s) to 21.9% (SSP585-2090s), whereas losses reach 75.52% in the Lvliang–Taiyue region mid-section under SSP585-2090s. This results in an overall northward contraction of the species’ climatic niche, a pattern consistent with observations made for other galliforms and montane passerines that exhibit upslope shifts and “habitat pinning” in response to warming. These findings align with the results obtained by Cao et al. [27], who projected habitat declines of 54.69–97.63% for the Brown Eared Pheasant in the Fen River Basin using MaxEnt modeling. In addition, centroid analysis confirmed that Shanxi Province remains the core of climatically suitable habitat despite projected shifts, reinforcing its critical importance for the long-term conservation of the species.

As a forest-dependent and range-restricted species, the Brown Eared Pheasant is confined to structurally complex mid-elevation deciduous and mixed broad-leaved forests [23,59]. Its limited dispersal capacity (approximately 5.7 km) and low genetic diversity constrain its ability to track shifting suitable habitats across an increasingly fragmented landscape [24,60]. Moreover, phenological shifts induced by warming, such as earlier budburst or peaks in arthropod abundance, may disrupt the synchrony between resource availability and breeding requirements [61]. Consequently, as documented for other dispersal-limited montane taxa, the actual colonization of newly suitable patches by the Brown Eared Pheasant is likely to lag behind climatic niche shifts, further intensifying overall range contraction [62,63].

The climate data used in this study represent the WorldClim baseline period (1970–2000), which may not fully capture recent climatic conditions under ongoing warming trends. Although most occurrence records were derived from recent surveys and observations, temporal mismatches between climate data and occurrence records may introduce uncertainty into habitat suitability predictions. Future studies incorporating multiple global climate models and temporally matched occurrence datasets could further improve habitat suitability assessments and better quantify uncertainty in future projections.

### 4.3. Recommendations for Conservation of the Brown Eared Pheasant

Systematic conservation planning provides a scientifically grounded framework for developing effective species protection strategies [64,65,66]. This study advances beyond previous distributional research by integrating Marxan and GAP analysis to translate habitat predictions into actionable conservation priorities. Our GAP analysis indicates that established reserves cover merely 27.73% of the identified priority zones. Most critical habitats remain completely unprotected, especially those located in the southern Lvliang and Taiyue Mountains (Shanxi Province), along with the Huanglong Mountains (Shaanxi Province). These regions may serve as both the current demographic core and areas with potential climate refugial value under continued warming trends. However, the species’ strong association with mid-elevation forests constrains its capacity to track suitable climatic conditions under future climate-warming conditions, creating a critical conservation challenge. Without timely intervention, the loss of connectivity between core populations and potential future refugial areas could severely compromise metapopulation persistence.

To secure the future survival of the Brown Eared Pheasant across China, we outline the following conservation strategies. (i) Expand the protected areas: adjust existing reserve boundaries and establish new conservation units in the southern Lvliang, Taiyue, and Huanglong Mountains to incorporate currently unprotected priority zones. (ii) Increase habitat connectivity: Restore degraded habitats and construct ecological corridors to improve functional connectivity among fragmented populations. Evidence from biodiversity-offset assessments further suggests that restoring habitat area alone may not recover functional connectivity, highlighting the need to incorporate dispersal processes and corridor connectivity explicitly into conservation planning [67]. (iii) Establish dispersal corridors through reintroduction: implement carefully designed reintroduction programs aimed at creating dispersal corridors between isolated populations, with the goal of facilitating gene flow and establishing a connected metapopulation structure in the future. (iv) Strengthen management measures: implement long-term monitoring programs and enforce strict regulations on logging, grazing, tourism, and poaching to maintain habitat integrity and reduce anthropogenic disturbance.

## 5. Conclusions

We combined species distribution modeling with systematic conservation planning to assess climate change impacts on the Brown Eared Pheasant and identify priority areas for conservation. Our results show that annual temperature range, precipitation-related variables, and elevation are the main factors determining habitat suitability. Future climate projections suggest a shift in habitat distribution, with considerable habitat losses under high-emission scenarios and the remaining suitable areas mainly occurring in northern and mountainous regions. Among the priority conservation areas identified by Marxan, 72.27% are located outside existing protected areas, indicating substantial conservation gaps. Conservation efforts for the Brown Eared Pheasant should focus on improving protection in identified gap areas, maintaining connectivity among fragmented populations, and incorporating future climate scenarios into conservation planning. These findings provide a scientific basis for developing climate-adaptive conservation strategies for this threatened mountain bird.

## Figures and Tables

**Table 1:** Dataset names, resolutions, and sources used in this study.

Dataset Name	Resolution	Data Source Website
Historical climate data	1 km × 1 km	Worldclim (https://worldclim.org/)
Future climate data	5 km × 5 km	Worldclim (https://worldclim.org/)
Elevation	1 km × 1 km	Worldclim (https://worldclim.org/)
Terrain ruggedness index	1 km × 1 km	Calculated based on DEM data in QGIS
Protected areas	/	Protected Planet (https://www.protectedplanet.net/)
Population density	1 km × 1 km	Worldpop (https://hub.worldpop.org/)
Road density	/	OpenStreetMap (https://www.openstreetmap.org/ accessed on 13 April 2025)
Global impervious surface	1 km × 1 km	Zenodo (https://zenodo.org/record/5220816 accessed on 13 April 2025)
land use	30 m × 30 m	National Catalogue Service for Geographic Information (https://www.webmap.cn/mapDataAction.do?method=globalLandCove accessed on 13 April 2025)

**Table 2:** Predictive performance of different modeling algorithms.

Model	AUC (Mean)	AUC (SD)	TSS (Mean)	TSS (SD)
ANN	0.7652	0.0086	0.6684	0.0189
CTA	0.7612	0.0083	0.6914	0.0232
FDA	0.7582	0.0011	0.7042	0.0141
GAM	0.8812	0.0044	0.6960	0.0055
GBM	0.8840	0.0017	0.8004	0.0046
GLM	0.8698	0.0011	0.8328	0.0573
MARS	0.7752	0.0011	0.6522	0.0073
MAXENT	0.8734	0.0053	0.7514	0.0052
RF	0.8422	0.0008	0.6974	0.0029
SRE	0.8464	0.0054	0.6928	0.0110
Ensemble Model	0.9520	0.0050	0.8328	0.0100

## Data Availability

The occurrence records of the Brown Eared Pheasant used in this study contain sensitive locality information for a nationally protected species and are therefore not publicly available. Access to these data may be granted upon reasonable request to the corresponding author.

## Supplementary Materials

The following supporting information can be downloaded at: https://www.mdpi.com/article/10.3390/ani16162475/s1, Table S1: Land use type and its corresponding LDI coefficients; Table S2: Nature reserves for the Brown Eared Pheasant included in the GAP analysis; Figure S1: Pearson correlation matrix of environmental variables.
