# Supplementary material for: Where Can a Threatened Pheasant Persist? Identifying Climate Change Refugia and Mapping Protection Gaps in China
Source: Animals (Basel). 2026 Aug 9;16(16):2475. doi: 10.3390/ani16162475 (PMC13508919; doi:10.3390/ani16162475)
Supplement: Supplementary file 1 [file animals-16-02475-s001.zip › animals-4434267-supplementary.pdf]

Supplementary Material

Table S1. Land use type and its corresponding LDI coefficients.

Land Use Type	LDI Coefficient
Cultivated land	4.54
Forest	1.58
Grass land	2.77
Shrubland	1.58
Wetland	1
Water body	1
Artificial surfaces	8.66
Bareland	6.92
Permanent snow and Ice	1

Table S2. Nature reserves for the Brown Eared Pheasant included in the GAP analysis

Number	Level	Province	Nature reserve
1	National	Beijing municipality	Baihuashan, BHS
2	National	Hebei Province	Xiaowutaishan, XWTS
3	Provincal	Hebei Province	Jinhuashan-Henglingzi, JHS-HLZ
4	National	Shanxi Province	Luyashan, LYS
5	National	Shanxi Province	Pangquangou, PQG
6	National	Shanxi Province	Wulushan, WLS
7	National	Shanxi Province	Heichashan, HCS
8	National	Shanxi Province	Lingkongshan, LKS
9	Provincal	Shanxi Province	Tianlongshan, TLS
10	Provincal	Shanxi Province	Lingjinggou, LJG
11	Provincal	Shanxi Province	fenheshangyou, FHSY
12	Provincal	Shanxi Province	Yundingshan, YDS
13	Provincal	Shanxi Province	Hanxinling, HSL
14	Provincal	Shanxi Province	Yunzhongshan, YZS
15	Provincal	Shanxi Province	Hejiashan, HJS
16	Provincal	Shanxi Province	Renzushan, RZS
17	Provincal	Shanxi Province	Weifenhe, WFH
18	Provincal	Shanxi Province	Tuanyuanshan, TYS
19	Provincal	Shanxi Province	Xuegongling, XGL
20	National	Shaanxi Province	Yanan Huanglongshan, YAHLS
21	National	Shaanxi Province	Hancheng Huanglongshan, HCHLS

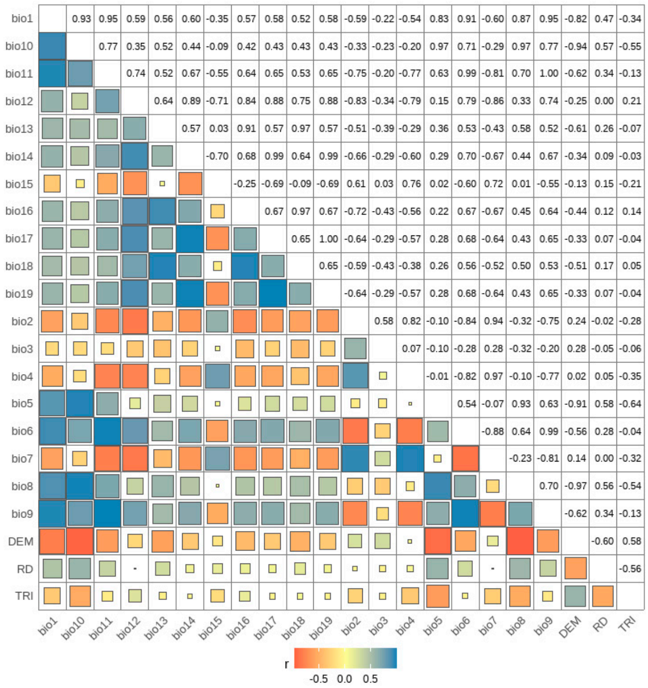

Figure S1. Pearson correlation matrix of environmental variables
